# Innovative granular formulation of *Metarhizium robertsii* microsclerotia and blastospores for cattle tick control

**DOI:** 10.1038/s41598-021-84142-8

**Published:** 2021-03-02

**Authors:** Allan Felipe Marciano, Gabriel Moura Mascarin, Renato Felipe Ferreira Franco, Patrícia Silva Golo, Stefan T. Jaronski, Éverton Kort Kamp Fernandes, Vânia Rita Elias Pinheiro Bittencourt

**Affiliations:** 1 Department of Animal Parasitology, Veterinary Institute, Federal Rural University of Rio de Janeiro, Seropédica, Rio de Janeiro, 23897-000 Brazil; 2 Laboratory of Environmental Microbiology, Brazilian Agricultural Research Corporation, Embrapa Environment, Jaguariúna, São Paulo, 13918-110 Brazil; 3 Institute of Tropical Pathology and Public Health, Federal University of Goiás (UFG), Goiânia, Goiás, 74690-900 Brazil; 4 Jaronski Mycological Consulting, Blacksburg, VA 24060 USA

**Keywords:** Biological techniques, Biotechnology, Industrial microbiology, Soil microbiology, Fungal biology, Fungal ecology, Fungal pathogenesis, Fungal systems biology

## Abstract

The tick *Rhipicephalus microplus* poses a serious threat to the cattle industry, resulting in economic losses aggravated by tick resistance to chemical acaricides. Strains of *Metarhizium* spp., a well-known group of entomopathogenic fungi, can contribute to managing this ectoparasite. We explored two novel granular, microsclerotia- or blastospores-based formulations of *Metarhizium robertsii* for *R. microplus* control under semi-field conditions. Fungal persistence in soil was also observed for 336 days. The experiment used pots of *Urochloa decumbens* cv. Basilisk grass, treated with 0.25 or 0.5 mg of granular formulation/cm^2^ (25 or 50 kg/ha) applied to the soil surface prior to transferring engorged tick females onto the treated soil. The fungal granules yielded more conidia with subsequent sporulation under controlled indoor conditions than in the outdoor environment, where the levels of fungus rapidly declined over time. *Metarhizium*-root colonization ranged from 25 to 66.7% depending on the propagule and rate. Fungal formulations significantly reduced the number of tick larvae during the humid season, reaching at least 64.8% relative efficacy. Microsclerotia or blastospores-granular formulations of *M. robertsii* can reduce the impact of *R. microplus*, and thus prove to be a promising tool in the control of ticks.

## Introduction

The effective use of microorganisms in biological control programs of arthropod pests depends particularly on the technology for mass production aligned with an effective formulation strategy^1^. Adverse environmental conditions, microbial competitors and host ecology are, however, possible limitations for the successful application of microbial bioproducts targeting either below- or above-ground environments^2^. Among entomopathogenic fungi, the genus *Metarhizium* is prominent in biological control of a broad range of agricultural pests and constitutes one of the most common active ingredients in mycoinsecticides and mycoacaricides marketed throughout the world^3–7^.

*Metarhizium* spp. are able to produce different types of propagules, such as conidia and blastospores (BLS)^8^, and some species may produce, by liquid fermentation, compact aggregates of melanized mass of hyphal threads known as microsclerotia (MS), which are structures more resistant to desiccation than fungal conidia^9^, which are important attributes for developing consistently effective bioproducts to control soil-dwelling arthropod pests. BLS are also produced by liquid fermentation, which is convenient for the industry in terms of reduced time, labor and cost^4^. Both MS and BLS are amenable to dry formulations and upon rehydration they can form infective conidia in situ when applied to the environment; in turn, the resultant conidia are able to disperse and infect arthropod hosts^4^. Of particular interest, *Metarhizium robertsii*, *M. brunneum* and *M. anisopliae* not only infect and kill a target pest, but can also colonize the rhizosphere of surrounding plants to their benefit^10^. Bio-formulations produced by encapsulation or granulation methods of liquid-culture-grown fungal propagules are gaining attention as an effective environmentally-friendly alternative to chemical insecticides and acaricides, as they can enhance fungal efficacy, improve field persistence, and extend product shelf-life. These propagules harbor great promise to replacing aerial conidia, which are still the standard used in fungal biopesticides^3^.

The soil is the site where *R. microplus* engorged females lay their eggs after dropping from the host, during its non-parasitic life phase, and such behavior is strongly influenced by environmental factors^11^. During this non-parasitic life stage, females, eggs and newly hatched larvae remain on the ground comprising 95% of the tick population present in the livestock system, which make them easily exposed targets to contact acaricides^12^. Therefore, the application of acaricides targeting eggs and larvae on the pastures would more effectively reach the tick population than applications on infested cattle and also prevent or minimize initial infestation levels on the host cattle. Despite the fact that fungal entomopathogens for tick control have been studied for more than two decades^13^, chemicals remain the chief method to control ticks particularly in Brazil.

Granular formulations for microbial agents are gaining special attention as a cost-effective technology for use with biocontrol agents or bioinoculants in agriculture^14^. When targeting soil-dwelling arthropod pests, small granules could be a viable option to deliver fungal propagules to sites occupied by the target pests. Dry MS-containing granules of *M. brunneum*, for instance, exhibited superior efficacy in controlling sugarbeet root maggots compared to aerial conidia produced on cereal grains^15^. With proper moisture conditions, the fungus in MS can germinate, grow out, and subsequently sporulate, releasing a large amount of infective conidia in situ where the pest is located. Unlike MS, BLS are environmentally sensitive yeast-like cells that have been tested in granular formulations to control ticks^16^, but a comparison between MS and BLS-based formulations for cattle tick control along with their persistence in soil has not been addressed until this study. Because BLS are more sensitive to drying, storage and adverse environmental conditions, the use of BLS are underexplored, especially regarding soil applications^17^. With technological advances in the production of BLS, these disadvantages are being overcome^17^ along with the selection of more resilient strains that are able to withstand environmental constraints^18^.

We investigated the efficacy of granular formulations containing either MS or BLS of *M. robertsii* strain IP 146 against the tick *R. microplus* in semi-natural pasture conditions. The granular formulation tested in this study was produced based on low-cost matrices of carriers containing MS or BLS for soil applications. We determined the fungal persistence in soil exposed to natural ambient conditions, and further assessed the root colonization by this fungus in the grass plant *Urochloa decumbens*, extensively employed for grass-fed cattle in Brazil.

## Materials and methods

### Experimental design

The study was carried out for 337 consecutive days (Supplementary Fig. S1), January to December, 2019. During this interval, two distinct climatic conditions were encountered: a period of hot days (27.24 ± 2.10 °C, mean ± standard deviation) and high volume of precipitation (9.52 ± 16.82 mm), and a second season with milder temperatures (22.12 ± 2.86 °C) and less rain (2.21 ± 5.08 mm). The semi-field trial was conducted at Federal Rural University of Rio de Janeiro—UFRRJ, in Rio de Janeiro state, Brazil (22° 45′ 54.9′′ South and 43° 41′ 57.2′′ West, 24 m a.s.l.). Climatic conditions were monitored using data from a meteorological station. *Urochloa decumbens* cv. Basilisk seeds (Wolf seeds, Ribeirão Preto, SP, Brazil) were sown in polypropylene pots (22 × 24 × 24 cm). The soil, a Planosol, was autoclaved two cycles at 120 °C, 20 min, to eliminate any wild *Metarhizium* spp., before sowing with seeds and conducting the actual trials, three months later. The temperature of the soil was recorded with an analog thermometer 5 cm deep in the soil from one pot for each group, daily after treatment. The pots were placed in groups following a completely randomized block design under direct incidence of sun and rain (Supplementary Fig. S2).

Based on previous tests (unpublished data), two concentrations of each granular formulation of microsclerotia (MS) or blastospores (BLS), were established: 0.5 mg/cm^2^ soil surface (50 kg/ha), subsequently termed MS-G 50 or BLS-G 50, and 0.25 mg/cm^2^ (25 kg/ha), termed hereafter MS-G 25 or BLS-G 25. There were five treatments with eight grass pots each. Two groups were treated with the granular formulation containing microsclerotia (MS) at each of the described rates. Two other groups were treated with each of the two rates of the granular formulation containing blastospores (BLS). The control group (CTRL) was treated with 0.5 mg/cm^2^ (50 kg/ha) of granules formulated without the fungus. The area of the soil surface in pots was 576 cm^2^, requiring 288 mg or 144 mg of granules per pot for the two rates, respectively.

### Granular formulations

The granular formulations, containing either MS or BLS of *M. robertsii* IP 146^19^, were produced at Federal University of Goiás – UFG.

BLS were produced in 250-mL flasks containing modified Adámek medium^20^ inoculated with a conidial suspension of 2 mL at 1.0 × 10^8^ conidia/mL, and agitated for 4 days^18^. Biomass was centrifuged and resuspended in 0.01% Tween 80 (Labsynth, Brazil) twice; BLS were quantified in a Neubauer chamber and concentration adjusted to 1.0 × 10^8^ BLS/mL. MS were grown in basal medium^21^ where 10 mL of the fungal suspension (5.0 × 10^7^ conidia/mL) were added to 90 mL medium, in 250-mL flasks. The cultures were agitated 4 days^15,22^ after which the biomass was centrifuged and resuspended in 0.01% Tween 80 twice. The MS concentration was determined according to Jaronski and Jackson^15^ (Supplementary Fig. S3).

Liquid biomass (1.0 × 10^4^ MS/mL or 2.0 × 10^8^ BLS/mL) was mixed with 950 mg of microcrystalline cellulose 101 (Mingtai Chemical, Taiwan) and 50 mg of Psyllium (Natural do Norte, Brazil) for each mL of biomass,  then the material was filtered through a 70-mm diameter filter paper (14 μm porosity; J.PROLAB, São José dos Pinhais, SP, Brazil) under vacuum, homogenized and passed through a 1-mm sieve. Psyllium husk comes from the crushed seeds of the *Plantago ovata* plant, a herb native to parts of Asia, the Mediterranean, and North Africa^23^. The resultant granules were dried in fluidized bed dryer (1000 × 1850 × 630 mm, 1.5 mm^3^/min) (model FBD 1.0, LabMaq, Ribeirão Preto, SP, Brazil) until final moisture reached 5 ± 1% (Supplementary Fig. S3). We obtained 80 g of granules with 1.25 × 10^4^ MS/g or 2.5 × 10^6^ BLS/g, which granules were further stored at 6 ± 1 °C in sealed 50-mL centrifuged tubes until use (eight days).

Formulation batches of fungal granules were evaluated for viability based on hyphal (myceliogenic) germination and sporogenic germination (conidial production)^8^ (Supplementary Fig. S4). Each granular formulation (7 mg) was sprinkled onto the surface of Petri plates (90 × 15 mm) containing water agar medium (2% w/v) or onto Petri plates (50 × 10 mm) containing 10 g of the same soil (sterile or non-sterile) from the pots. Petri plates were incubated at 25 ± 1 °C for seven days and then examined for hyphal germination and conidial production. The viability of conidia was determined by plating suspensions on CTC medium and assessing the number of colony-forming units (CFUs)^24^. Each condition was performed in triplicate and the test was independently repeated at least twice.

### Efficacy for *Rhipicephalus microplus* in semi-field test

The soil in the grass pots was treated by spreading the granules manually as evenly as possible over the surface of previously irrigated soil, and the fungal sporulation was monitored daily. Prior to using in the semi-field trials, we disinfected the cuticles of engorged female ticks^25^; the females were then weighed individually and separated into five classes according to their weights. Forty groups of five females (one tick from each class) were placed on the soil of each grass pot. The first evaluation started with the introduction of engorged females into the pots 8 days after the first fungal application onto the soil (= day 0). In a second evaluation,176 days after the first treatment, engorged females were exposed to the soil to evaluate a possible residual effect from the first treatment. Subsequently, 218 days after the first treatment, a new fungal application was performed (second fungal application) and a third population of engorged females was exposed to the treated soil (third evaluation). After incubation of engorged females on the treated soil, daily inspections were carried out to monitor oviposition and cumulative mortality. As larvae hatched and climbed to the top of the grass blades (Supplementary Fig. S2), they were recovered and quantified to calculate a relative efficacy (RE)^25^. Fungi from dead engorged females were isolated as described in the literature^25,48,49^.

### Isolation of fungi

To assess the persistence of *M. robertsii* in the soil, CFUs levels were monitored in soil samples collected one day before the first treatment (day -1) and on days 8, 15, 30, 176, 210, 225, 232, 246, and 336 after the first treatment. Three soil samples from each pot were collected and processed for fungal isolation per the method of Fernandes, et al.^26^.

On days-1, 30, 176, and 336, three *U. decumbens* plants were removed carefully from the soil of each grass pot. Three pieces of each plant’s roots (approx. 0.6 mm in length)^27^ were plated on CTC medium after surface sterilization, and incubated at 25 ± 1 °C for 21 days. The success rate in colonizing *U. decumbens* roots was calculated by counting the number of plants with positive root colonization by *M. robertsii* outgrowth, confirmed based on microscopic examination^28^.

### Statistical analysis

All analyses were performed with R Core Team, version 4.0.2 ^29^. Tick larval counts were fitted to a generalized linear mixed model (GLMM) with negative binomial distribution (log link function), with the package “glmmTMB”^30^, including tick female exposure (infestation), treatment (fungal formulations and control) and their interaction terms as fixed effects, and block as random effect. Relative efficacy data were fitted to linear mixed model with Gaussian distribution, including evaluation period, formulation and their interaction as fixed factors and block as random effect. Tick survival was analyzed by Kaplan–Meier Survivorship, and survival curves were compared with log-rank test (*P* < 0.05) (package “survival”)^31^. Successful colonization of roots by *M. robertsii* was fitted to GLMM with binomial distribution (logit link), including treatment as fixed effect.

Fungal viability of dry granules was fitted to a GLMM with binomial distribution and logit link function. Conidial production was log_10_-transformed and fitted to a linear mixed model with Gaussian distribution. In both models, fixed effects were attributed to propagule type, substrate type, and their interaction, and observational level and/or experimental date as random effects.

Normally distributed observations with heterogeneous variance in the number of CFUs/g soil were fitted to GLM using the function generalized least square (gls) in “nlme” package^32^, implemented separately for the first fungal application during summer season and the second fungal application during winter season. The model included treatments, evaluation date, and their interaction term as fixed effects, while heterogeneous variance was attributed to each treatment × time. Correlation between climatic factors recorded during the semi-field experiment and fungal persistence in the soil was performed with non-parametric Spearman rank method. All pairwise multiple comparisons of treatment means were performed with Tukey HSD test (*P* < 0.05), implemented with “emmeans” package^33^.

### Ethics statement

Engorged female ticks were obtained from a colony maintained on artificially infested calves in accordance with the regulations of the UFRRJ's Ethics Committee for Animal Experimentation, approved by the protocol # 9714220419 (Supplementary information). The access to Brazilian genetic heritage was approved by SisGen, protocol # A420934.

## Results

### Conidial yield and fungal viability in granular formulations

The viability of the granules and their conidial production were evaluated under different conditions of substrate and sterility, as well as the viability of the conidia obtained. Granules containing MS or BLS clearly showed the first visible hyphal projections or myceliogenic germination 2 days after incubation on water agar medium. In contrast, on sterile and non-sterile soil, hyphal growth occurred only 3 and 5 days after incubation, respectively. The granules on water agar and on sterile soil underwent conidiogenesis earlier, i.e., 5 days after incubation in comparison to the granules incubated on non-sterile soil, where sporulation did not take place until the 7th day.

Conidial production by granular formulations was strongly affected by the type of substrate and type of fungal propagule (interaction: *F*_2,29_ = 55.04, *P* < 0.0001). For both types of fungal propagule, sporogenic germination of *M. robertsii* IP 146 was always higher on granules inoculated on water agar medium followed by the sterile soil substrate, whereas lower conidial yields were attained by the granules incubated on non-sterile (natural) soil, regardless the propagule type (Table 1).

**Table 1:** Conidial yield (sporogenic germination) by granular formulations of microsclerotia (MS) or blastospores (BLS) of *Metarhizium robertsii* (IP 146 strain) on different types of substrates.

Propagule type	Substrate type	Conidia/g of granules		
MS	Sterile soil	153 × 10^8^ ± 6.41 × 10^8^	b	
	Non-sterile soil	11.9 × 10^8^ ± 0.50 × 10^8^	c	*
	Water agar	671 × 10^8^ ± 282 × 10^8^	a	
BLS	Sterile soil	143 × 10^8^ ± 6.01 × 10^8^	b	
	Non-sterile soil	5.83 × 10^8^ ± 0.24 × 10^8^	c	*
	Water agar	612 × 10^8^ ± 25.7 × 10^8^	a	

Means (± SE) in the same column followed by different lowercase letters are significantly different among substrate types, and asterisk (*) indicates significant difference between MS and BLS within each substrate type. Pairwise multiple comparisons were conducted with the Tukey HSD test at *P* < 0.05.

MS granules produced considerably greater numbers of conidia than BLS granules when inoculated on non-sterile soil, and this superiority was more pronounced when incubated on water agar medium (Table 1). Viability of conidia produced by MS and BLS granules reached ≥ 85% and was strongly affected by the substrate type (*χ*^2^_2_ = 77.82, *P* < 0.0001), irrespective of propagule type; low viability rates occurred when granules were placed on non-sterile soil (Supplementary Fig. S5). MS granules produced 1.07 ± 0.07 × 10^7^ and 8.39 ± 0.67 × 10^5^ conidia/g sterile and non-sterile soil, respectively. The BLS granules reached 1.01 ± 0.06 × 10^7^ in sterile soil and 4.13 ± 0.66 × 10^5^ in non-sterile soil.

### Efficacy of granular bio-formulations against *R. microplus* in semi-field trials

**Figure 1:**
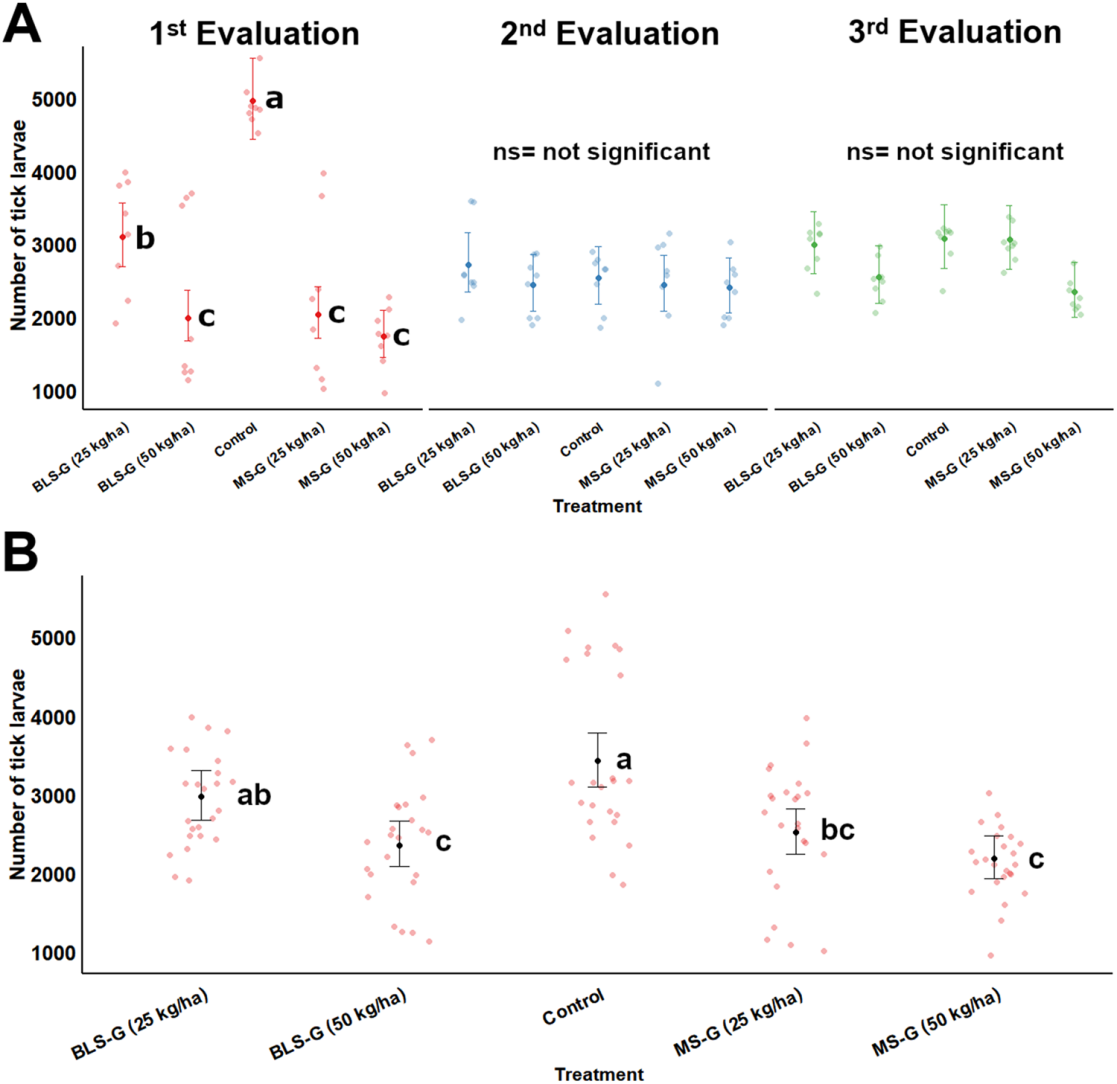
Impact of granular formulations at different rates of *Metarhizium robertsii* microsclerotia and blastospores on the density of tick larvae on grass pots. Number of *Rhipicephalus microplus* larvae counted on grass-pots after soil amendments with 50 or 25 kg/ha of microsclerotia-based granules (MS-G) or blastospores-based granules (BLS-G). (**A**) The average number of larvae compared between treatments in three evaluations periods when *R. microplus* female ticks were exposed to the treated environment. In the first and second evaluation, the immediate and residual effect of the first fungal soil application was assessed respectively, in the third evaluation the effect of a new application of the formulations was verified. (**B**) Overall effect obtained in the number of tick larvae on the three populations of ticks throughout the test period considering the two fungal treatments. Means (n = 8) are dots and whiskers represent 95% confidence intervals. Means followed by distinct letters indicate significant differences (Tukey HSD test *P* < 0.05). Raw data are shaded dots.

During the tests, two fungal applications and three evaluations were performed on the tick population, of which two evaluations took place after the first fungal application and a third evaluation was done after the second soil application of the fungal granules. Both concentrations of granular formulations containing MS or BLS significantly decreased the number of tick offspring (reduced numbers of larvae attached to the grass leaves) during the first evaluation period after the first fungal application (*χ*^2^_8_ = 78.83, *P* < 0.0001), relative to the control group (Fig. 1A). After the first fungal application, significant reductions in the number of larvae were achieved with 50 kg/ha of BLS-based granules and 25 kg/ha of MS-based granules (MS-G 0.25). Conversely, the treatment with 25 kg/ha of BLS-based granules (BLS-G 0.25) was not as effective as the other treatments, although it still presented significantly fewer larvae than the control group (Fig. 1A). After five months from the first treatment, a second population of female ticks (second evaluation) was placed into the pots and all groups had a similar number of larvae and no residual effect of *M. robertsii* IP 146 was observed (*P* > 0.05, Fig. 1A). A second soil treatment took place followed by a third evaluation of tick population in a different season of the year under a colder and drier climate, but this new treatment with granular formulations was insufficient to reduce larvae outbreak in the grass pots when compared to the control group (*P* > 0.05, Fig. 1A).

Nevertheless, when clustering the data across both fungal applications along with the three evaluation periods, *M. robertsii* formulations negatively affected larval load in the pasture (*χ*^2^_4_ = 42.77, *P* < 0.0001) (Fig. 1B). The mean number of tick larvae was lower in the pots treated with the high rate of MS-G (*P* < 0.0001) and BLS-G (*P* = 0.0001), as well as with the lower rate of MS-G (*P* = 0.0012), in comparison to the control group. The treatment with BLS-G (*P* = 0.3305) did not differ from the untreated control, but it also had similar effect from that caused by MS-G 25 *P* = 0.2258) (Fig. 1B).

There was a significant interaction effect between application and evaluation period regarding the relative efficacy against tick larvae (*χ*^2^_6_ = 13.23, *P* = 0.0395). The greatest relative efficacy was achieved in the first exposure of females to the fungus-treated soil (first evaluation), with efficacies of 38.4% and 64.6% by BLS-G 25 and MS-G 50, respectively (Table 2). Without reapplying the fungal formulations to grass pots, the relative efficacy was substantially reduced on the second evaluation period in all treatments in comparison to the control groups, with no significant differences among treatments (*P* > 0.05), and averages ranged from 4.1% (BLS-G 25) to 12.4% (MS-G 25). After the second application of fungal granules, we documented a pronounced increase in the relative efficacy for the third population of ticks exposed to MS-G 50 in contrast to MS-G 0.25, and the mean relative efficacy across treatments varied between 1.7 and 23.9% (Table 2).

**Table 2:** Relative efficacy (%) of tick control obtained in three evaluations periods after exposure of *Rhipicephalus microplus* population to soil treatment with 25 or 50 kg/ha of granular formulations containing *Metarhizium robertsii* microsclerotia (MS-G) or blastospores (BLS-G). In the first and second evaluations, the immediate and residual effect of the first fungal soil application was assessed, respectively, while in the third evaluation the effect of a new application of these fungal treatments was recorded.

Evaluation period	Treatment group (kg/ha)	Relative efficacy (%)*	95% CI**		
1st Evaluation	MS-G 50	64.8	54.84, 74.7	A	a
	MS-G 25	56.1	46.14, 66.0	A	a
	BLS-G 50	55.8	45.86, 65.7	A	a
	BLS-G 25	36.3	26.34, 46.2	B	a
2nd Evaluation	MS-G 50	10.8	0.88, 20.7	A	b
	MS-G 25	12.4	2.51, 22.3	A	b
	BLS-G 50	8.7	-1.18, 18.6	A	b
	BLS-G 25	4.1	-5.77, 14.0	A	b
3rd Evaluation	MS-G 0.5	23.9	13.97, 33.8	A	b
	MS-G 0.25	1.7	-8.18, 11.6	B	b
	BLS-G 0.5	18.8	8.92, 28.8	AB	b
	BLS-G 0.25	7.1	-2.79, 17.0	AB	b

(*) Means (n = 8) in the same column followed by different uppercase letters are significantly different between treatment groups within the same evaluation period. Means (n = 8) in the same column followed by different lowercase letters are significantly different comparing the same treatment group among evaluations periods. Pairwise multiple comparisons were conducted with Tukey HSD test at *P* < 0.05. (**) Confidence level.

During the three exposures of ticks to fungus-treated soil, mortality of engorged females was recorded only after the oviposition period (natural death). Thus, there was no significant effect of *M. robertsii* formulations on tick female survival rates (*χ*^2^_4_ = 0.88, *P* = 0.95) (Supplementary Fig. S6). Only filamentous fungi other than *Metarhizium* spp. were isolated from tick cadavers found during the experimental period. Thus, the major effect was  found on survival of larval ticks.

### Fungal persistence in the soil and re-isolation from roots

**Figure 2:**
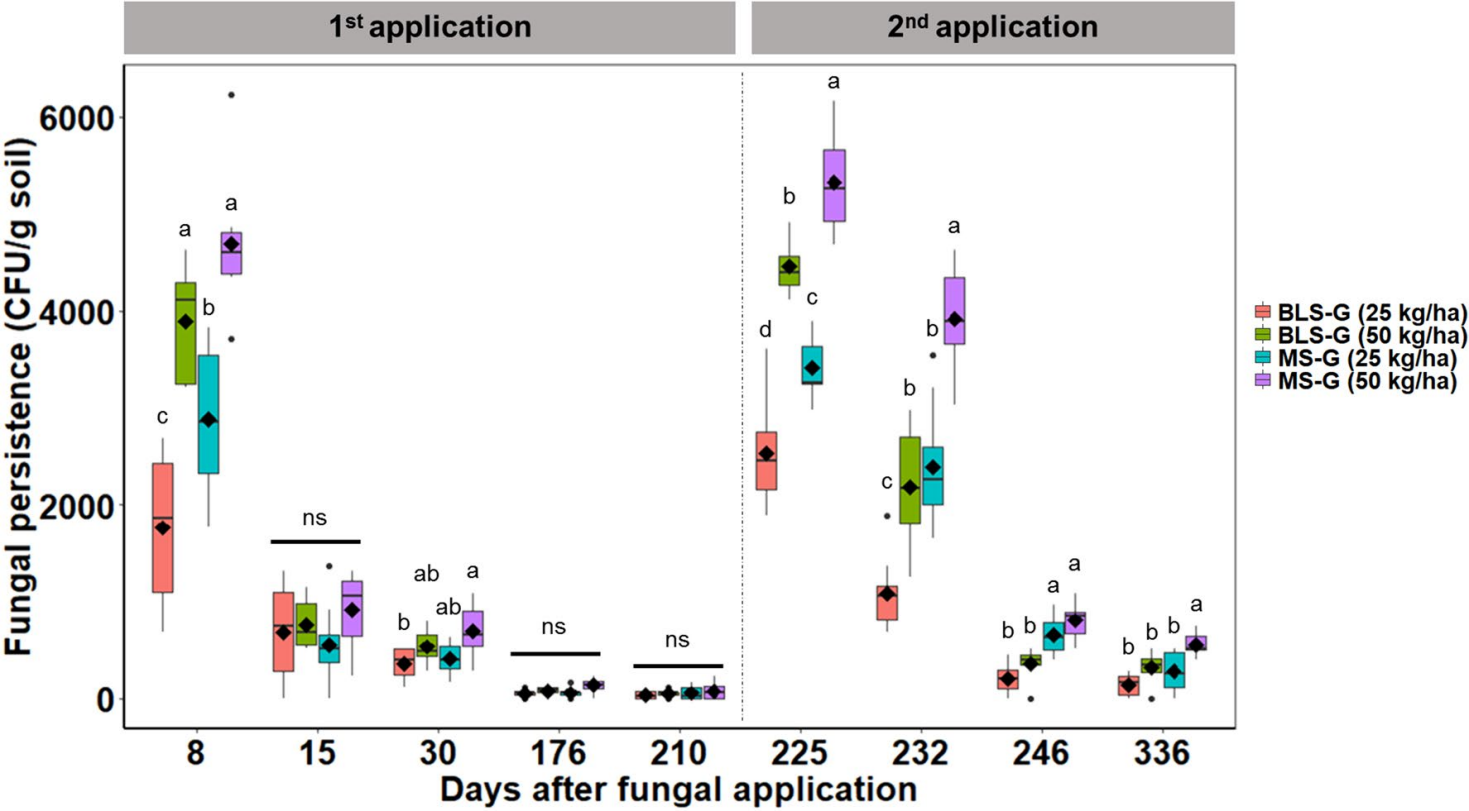
Fungal persistence in the soil of pots treated with *Metarhizium robertsii* granular formulations. Box plot of *M. robertsii* colony forming units (CFUs) per gram of soil obtained after the 1st and 2nd fungal application under field conditions. Boxes show the median, 25th and 75th percentiles, while error bars show 10th and 90th percentiles. Means (n = 8) are black diamonds and followed by distinct letters indicate a significant difference between treatments (Tukey HSD test *P* < 0.05). BLS-G: granular blastospores formulation; MS-G: granular microsclerotia formulation.

Before and after the treatment of the *U. decubens* pots with the fungal granules, periodic collections of soil and roots of the plant were made for the evaluation of the fungal persistence in the environment. In all treatments, *Metarhizium* spp. were not recovered from soil or root samples before the application of fungal formulations. After applying the fungal granules to the pots, it was possible to retrieve *M. robertsii* IP 146 from the soil of all fungal treatments over the experimental span (Fig. 2). There was no recovery of *Metarhizium* spp. from the soil samples of the untreated controls.

The highest number of CFUs obtained in both seasons was in the first soil sampling soon after the first and the second fungal applications (day 8 and day 225) (Fig. 2). After the first fungal application, during the summer season, the greatest number of CFUs produced by the fungal granules coincided with the highest temperatures and global radiation index registered (Supplementary Fig. S7A), resulting in a positive correlation. On the other hand, the number of CFUs abruptly declined as time progressed (Fig. 2); a high cumulative rainfall occurred, accompanied by high RH indices. CFUs numbers had a negative correlation with these two abiotic variables (Supplementary Fig. S7A). Similarly, the levels of *M. robertsii* IP 146 invariably decreased after the second fungal application of all fungal formulations tested, but at a slower trend compared with the first fungal application (Fig. 2). The second fungal application was performed on colder days during the winter, which also coincided with the highest number of CFUs of *M. robertsii* recorded during the experimental timeline (Fig. 2). There was a substantial negative correlation between CFUs numbers and the ambient minimum temperature (Supplementary Fig. S7B). Despite the cooler weather in the second experimental period, soil temperatures measured in the grass pots were higher at the beginning of the evaluation than at the end, which resulted in a positive correlation with the high number of CFUs (Supplementary Fig. S7B). After the first fungal application (from day 8 to day 210), soil levels of *M. robertsii* significantly declined, varying among the fungal formulations. There was a significant interaction effect between treatments and soil sampling dates on fungal persistence (CFUs numbers over time) (*F*_12,140_ = 6.66, *P* < 0.0001). After the first fungal application, on day 8, the number of CFUs obtained with the high dose of fungal granules per cm^2^ (MS-G 50 and BLS-G 50) significantly exceeded the MS-G 25 and BLS-G 25 formulations. Moreover, the soils treated with MS granules at the lower rate (MS-G 0.25) had significantly more CFUs than those treated with the corresponding rate of BLS granules (Fig. 2), reflecting the production of conidia by the MS. Levels of fungus CFUs markedly decreased 15 days after the first fungal application and equaled the number of CFUs across all treatment groups (Fig. 2). In the following evaluation (day 30), the MS-G 50 treatment presented higher CFUs numbers in the soil than the BLS-G 25 treatment (*P* = 0.0387), even with a declining number of CFUs. The BLS-G 50 and MS-G 25 treatments exhibited similar CFUs numbers and did not differ from the other treatments. In the fourth (day 176) and fifth soil sampling (day 210), the number of CFUs per gram of soil in all treatment groups had significantly decreased in relation to the previous soil samplings, but were statistically similar between these two sampling points (Fig. 2).

Likewise, during the following experimental period, after the second fungal application (from day 225 to day 336), *M. robertsii* levels in pots progressively declined at different rates for the different fungal formulations. There was a significant interaction effect between applications and soil sampling dates regarding the fungal persistence in the soil (*F*_9,112_ = 24.47, *P* < 0.0001). On day 225, seven days after the second fungal application, the highest number of *M. robertsii* CFUs was achieved with the highest dose of granules tested (MS-G 50 and BLS-G 50), followed by the treatment with MS-G 0.25, which had more *M. robertsii* isolated from the soil than the BLS-G 25 group (Fig. 2). On day 232, the MS-G 50 group presented more CFUs of *M. robertsii* in the soil than the groups MS-0.25 (*P* = 0.0005), BLS-G 50 (*P* = 0.0001) or BLS-G 25 (*P* = 0.0001). The BLS-G 50 and MS-G 25 yielded similar CFUs per gram of soil and exhibited significantly more *M. robertsii* CFUs than the group treated with BLS-G 0.25, with *P* = 0.0023 and *P* = 0.0012 respectively. In the following soil collection (day 246), CFUs were more numerous in soil treated with MS granules than in those treated with BLS granular formulation; MS-G 25 treatment was significantly different from BLS-G 25 (*P* = 0.0006) and BLS-G 50 (*P* = 0.0250), as well as MS-G 50 treatment presented more CFUs per gram of soil than BLS-G 25 (*P* = 0.0001) and BLS-G 50 (*P* = 0.0001). In the last assessment (day 336), the MS-G 50 group had significantly higher CFUs per gram of soil than the groups MS-0.25 (*P* = 0.0165), BLS-G 50 (*P* = 0.0154) and BLS-G 25 (*P* = 0.0001), which did not differ from each other (Fig. 2).

**Figure 3:**
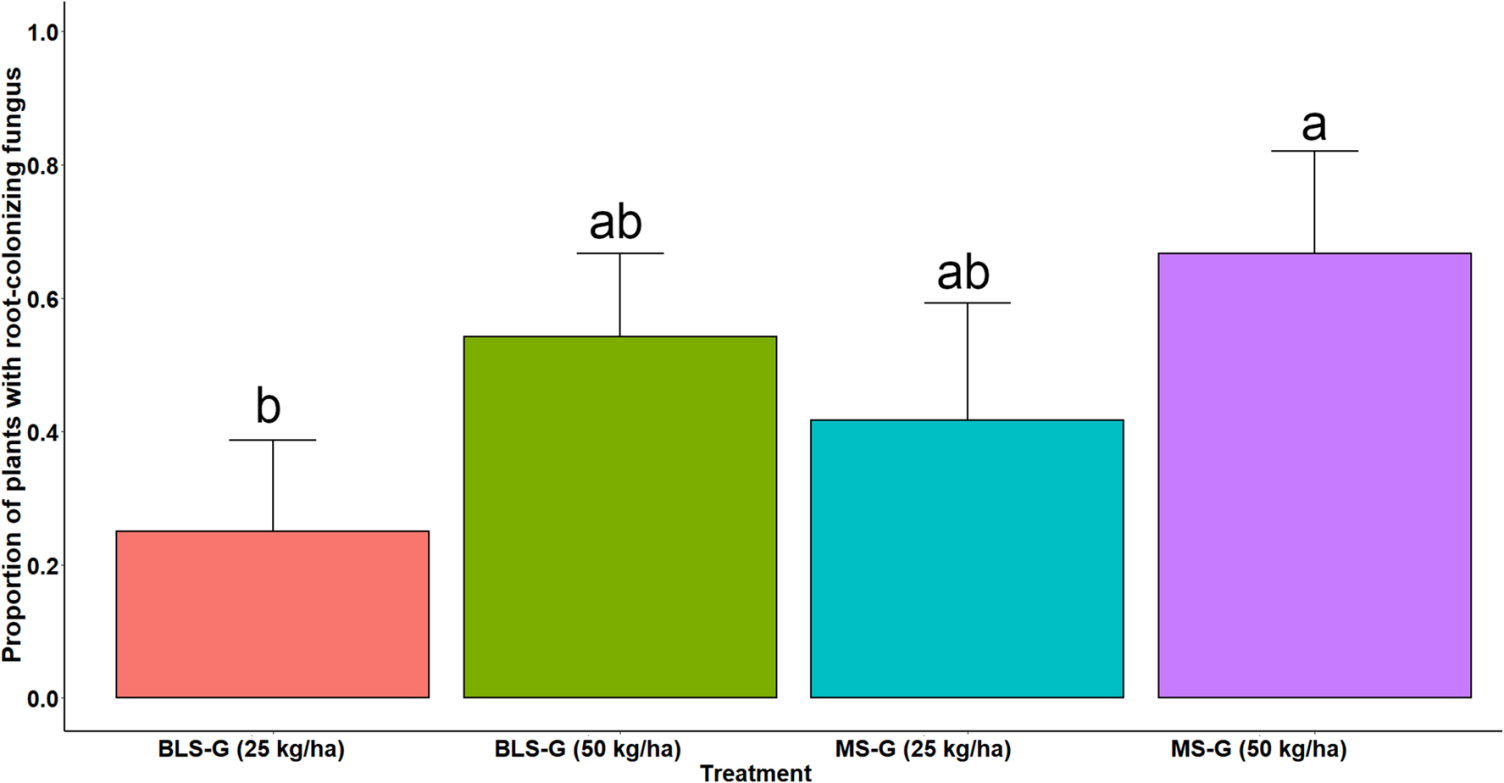
Effect of granular formulations of *Metarhizium robertsii* on root colonization of potted grass during field experiment of one year. Successful rate in root colonization at the end of the experiment (day 336) after soil amendment with granules containing microsclerotia (MS) or blastospores (BLS) of *M. robertsii* in granular formulations and applied at two doses (50 and 25 kg/ha). Control group had no root colonization by any naturally-inhabitant *Metarhizium* species.

Regarding *M. robertsii* recovery from *U. decumbens* roots, samples from all groups collected before (day—1), and after the first fungal application (day 30) had no fungal growth detected in surface-sterilized roots. Although *M. robertsii* was aseptically isolated from *U. decumbens* roots after the second fungal application, at the end of the experiment (day 336), no *Metarhizium* sp. was recovered from roots in the control pots. The only difference in the proportion of grass plants with root-colonizing *M. robertsii* among treatments was detected with the higher rate of MS granules (MS-G 50) resulting in 66.7 ± 9.6% (mean ± standard error [SE]) of plants with colonized roots, significantly higher than the treatment with BLS-G 25, which yielded only 25.0 ± 8.8% of plants positive for root colonization (*χ*^2^_3_ = 9.46, *P* = 0.0238). The additional fungal treatments (BLS-G 50 and MS-G 25) did not differ in their root colonization success in relation to the other treatments, and their incidence ranged from 41.7 to 54.2% (Fig. 3).

## Discussion

A large body of literature has supported the use of fungal entomopathogens, like *Metarhizium* spp. and *Beauveria* spp., to control ticks and has claimed the importance of developing formulation technologies that improve their effectiveness, persistence in the environment and ease of application^4,13,34,35^. The present study reports the soil amendment of *M. robertsii* granular formulations targeting pastures infested with *R. microplus* ticks under semi-field conditions. Long-term persistence of MS and BLS granules applied to the soil was also associated with the ultimate reduction in the number of tick larvae in grass pots.

Even though *M. robertsii* was not re-isolated from tick cadavers due to overgrowth by opportunistic saprobic fungi, the reduction of larvae from fungal treatments is presumed by sub-lethal infection of females which possibly reduced their egg production; in fact, the low egg production from *Metarhizium*-infected *R. microplus* females is well documented in the literature^18,36–39^. We also believe that the fungus may have infected eggs and larvae, which are more susceptible life stages than adult ticks^40^. In respect to the application of *Metarhizium* spp. in pasture environments and their control of *R. microplus* as well as other tick species, the results reported here corroborate previous studies^1,25,37,40–42^ which also showed relative success in controlling this non-parasitic phase of this cattle tick species.

The granular formulation composition, which contains a low-cost matrix of carriers, provides higher viability rates coupled with subsequently higher yields of conidia from both MS and BLS than calcium alginate beads earlier reported for encapsulation of *M. pemphigi* BLS^16^. In our study, both mycelogenic and sporogenic germination was notably affected by the nature of the substrate, especially when fungal granules were applied on natural (non-sterile) soil cultivated with grass under semi-field conditions versus water agar or sterile soil, with the aim of understanding possible abiotic factors affecting fungal germination and sporulation. MS and BLS granules showed fastest germination rates and greatest conidial production when incubated on water agar medium, which provides the optimal conditions for fungal growth, especially because of the sterile and high humidity microcosm. The high conidial yield achieved with water agar medium may be related to its high, consistent moisture and sterile substrate without competitors, which ensures a steady microclimate when compared to sterile or non-sterile soil substrate in outdoor pots since many field environmental factors can impact the germination of fungal structures^43^.The heterogeneous physical–chemical characteristics of the soil and the presence of microbial competitors, which are part of the resident microbiota in non-sterile soil, may have also lowered the myceliogenic germination, production of conidia and, consequently, the establishment of the strain IP 146 in this environment^44–47^. Even initially, with autoclaved soil, after planting the grass in the pots and exposing them to the outdoor weather conditions, a microbial community re-established itself slowly, but to a lower level than regular non-sterile soil; this was confirmed by the isolation of filamentous fungi other than *Metarhizium* from the soil samples inoculated on semi-selective CTC medium. The re-established microbial community in the grass-potted soil may have competed with *M. robertsii* for the same ecological niches. We suggest that the viability and inoculum production of fungal granules are strongly dictated by the environmental conditions and the status of the soil substrate where they were applied, thus impacting on the fungal development and persistence in less controlled and non-homogenous conditions. This situation is confirmed by comparing the concentration of conidia (CFUs) per g of soil obtained in the laboratory and in semi-field conditions in the first soil collection after fungal application. Despite the fact that BLS are considered environmentally sensitive structures less adapted to the soil^18^, and MS comprise resistant-like propagules more suitable for the control of soil-inhabiting arthropods in this environment^9,21^, the BLS granular formulation tested here enabled myceliogenic and sporogenic growth similar to the MS granules. In non-sterile soil, however, MS granules had a significantly higher conidial yield than BLS granules, which suggests a possible advantage of the former fungal propagule to cope with the soil fungistasis in comparison to the latter. This result also corroborates the evidence of natural resilience of *Metarhizium* spp. MS to adverse abiotic conditions, as previously reported by Jaronski and Jackson^15^. Despite this, it has been observed that, depending on the *Metarhizium* spp. strain, BLS may be more UV-B tolerant than other propagules^48^. The production of BLS in granular formulations would still be of value, considering their production yield and speed in liquid fermentation but future investigations are necessary to validate the efficiency of their granules.

Our outdoor semi-field experiment revealed a significant decline in fungal persistence in soil over time, an expected behavior for entomopathogenic fungi^49^. This phenomenon is influenced by intrinsic, edaphic, biotic, cultural, and climatic factors^49^. Several climatic variables were recorded during the long-term course of our semi-field experiment, and we observed that the two seasons, in which fungal applications took place, diverged in terms of fungal persistence and efficacy against the cattle tick. High temperatures combined with high incidence of rainfall marked the period after the first fungal application, during which the fungi had moderate tick efficacy (36–65%), and a severe decrease in soil levels of the fungus. During the second application, temperatures were mild, coupled with reduced precipitation, as expected for this locality of Brazil at the onset of winter, and the relative efficacy of tick control was greatly reduced (1.7–23.9%), but a slower decrease in CFUs/g soil during the initial 7 days after fungus application was recorded (Table 2).

The fungus benefited from the rainy season, with suitable soil moisture for its development, even under high global solar radiation, an important deleterious environmental factor to fungal survival in the environment^50^. Because the pots were covered with grass, the vegetation possibly blocked the direct solar radiation towards the fungal granules deposited on the soil surface, functioning as a buffer from extreme short-term changes in the microclimate and favoring the efficacy of *M. robertsii*^51^. The positive correlation of fungus levels in the soil with the values of temperature, as well as the negative correlation with relative humidity and cumulative rainfall occurred due during the transition from summer to autumn (Supplementary Fig. S7A), in which the days immediately after fungal application and with greater conidial production did not coincide with the climatic factors recorded. After the second fungal application, temperatures and rainfall were significantly reduced. The lack of rain made the soil drier and may have directly precluded propagule germination, sporulation, consequently affected fungus performance. While moisture content in the soil influences the sporulation of granules and interferes with the conidial survivorship^52,53^, moisture also plays an important role in the infection process of the fungus^46,54^.

Similar interpretations regarding the correlation between the density of the fungus and climatic factors must also be considered in the second fungal application. We observed a negative correlation between CFUs density and the temperature by the fact that the fungal granules produced more conidia shortly after application, but then fungal persistence substantially decreased in the transition from winter to spring season (warmer and wetter conditions) (Supplementary Fig. S7B).

In the subsurface soil, the longevity of the fungus is related to the ability of this microorganism to establish beneficial relationships with the plant rhizosphere^55^. Here, it was possible to re-isolate *M. robertsii* IP 146 from grass roots in all fungal treatments after 336 days of the first soil application. The fate of *Metarhizium* spp. in deeper regions and close to the roots after the inoculum density decreases in the soil surface has been reported by previous studies^56,57^. Accordingly, our study aligned with others to indicate there is a tendency for the fungus to migrate from the most superficial parts to deeper layers and closer to the root system after application to the soil, suggesting that *M. robertsii* IP 146 switches lifestyle from pathogen to endophyte in order to survive and persist in the soil habitat. Fungal granules may allow not only the superficial colonization of the soil but also in deeper layers occupied by the plant rhizosphere, extending the fungal persistence in agricultural ecosystems, as documented before^58^. In fact, *M. robertsii* has already been reported as preferably colonizing pastures and arable lands, possibly due to its wide adaptive skills to such ecological niche^59^. Although we cannot predict how the endophytic root colonization by *M. robertsii* could induce new infections to tick females and their egg masses on the soil surface, this behavior indeed contributes to fungal persistence and probably leads to new foci of infective inoculum derived from sporulated cadavers of other susceptible soil-dwelling arthropod hosts. This hypothesis will remain open but is currently under investigation by our group, as this fungus species is also an important pathogen to several soil-inhabiting insects that are root feeders, such as subterranean termites, wireworms, grubs, and sugarcane spittlebugs in Brazil.

Despite limited fungal persistence in face of environmental challenges, our fungal granular formulations provided a level of tick control. This efficacy was more pronounced after the first fungal application, when the treatments with MS-G at 50 kg/ha, MS-G at 25 kg/ha and BLS-G at 50 kg/ha diminished greater than 50% of tick larvae during the hotter and wetter season. In contrast, a weaker response was observed with BLS-G at 25 kg/ha, possibly due to the lower resilience of the BLS associated with its lower application rate, which did not yield the sufficient numbers of conidia to reduce tick density. Possibly, an increase of fungal biomass in the granular formulations could improve conidial yields in soils^60^.

Another important observation regarding the residual activity of the fungal formulations on a subsequent tick generation, without reapplication of granules. In our study control of the subsequent generation failed, pointing to the need of a more persistent bioproduct. With the second fungal application, only low efficacy was observed, even with lower temperature and radiation, conditions thought to be favorable to the fungal entomopathogen. It is suggested that, in this case, the scarce rainfall during the winter season may have decreased the ambient humidity (data not shown), thus altering the soil microclimate beneath the grass canopy and affecting the success of the fungal infection on ticks^46,54^.

Our findings highlight for the first time a strategy of using dry granular formulations consisting of either BLS or MS of *M. robertsii* for soil application to suppress *R. microplus* populations by the tick’s population fitness, as well as revealing the soil persistence and grass root-colonization of this fungus in the soil cultivated with grass. Our results advance knowledge on new cost-effective ways to use otherwise environmentally sensitive BLS for soil application and expand the usefulness of MS granules as effective alternative tools to chemical acaricides. This formulation technology can be easily scaled-up to industrial production of fungal propagules (MS and BLS), and has the advantage of inexpensive carrier ingredients. Future challenges to be tackled could focus on the extended fungal persistence after application under a range of conditions beyond the Brazilian situation, and to optimize granule rates applied to the soil with the aim to achieve higher efficacy rates in tick control.

## Supplementary Information

Supplementary Information

## Data Availability

The datasets generated during and/or analyzed during the current study are available from the corresponding author on reasonable request.
