# Supplementary material for: Innovative granular formulation of Metarhizium robertsii microsclerotia and blastospores for cattle tick control
Source: Sci Rep. 2021 Mar 2;11:4972. doi: 10.1038/s41598-021-84142-8 (PMC7925645; doi:10.1038/s41598-021-84142-8)
Supplement: Supplementary file 1 — Supplementary Information [file 41598_2021_84142_MOESM1_ESM.pdf]

Innovative granular formulation of *Metarhizium robertsii* microsclerotia and blastospores for cattle tick control

Allan Felipe Marciano[1,*], **Gabriel Moura Mascarin**[2,*], **Renato Felipe Ferreira Franco**[3], **Patr²cia Silva Golo**[1], **Stefan T. Jaronski**[4], **£verton Kort Kamp Fernandes**[3], **V©nia Rita Elias Pinheiro Bittencourt**[1,*]

[1] Department of Animal Parasitology, Veterinary Institute, Federal Rural University of Rio de Janeiro, Seropédica, Rio de Janeiro 23897-000, Brazil.

[2] Laboratory of Environmental Microbiology, Brazilian Agricultural Research Corporation, Embrapa Environment, Jaguariúna, São Paulo 13918-110, Brazil.

[3] Institute of Tropical Pathology and Public Health, Federal University of Goiás (UFG), Goiânia, Goiás 74690-900, Brazil.

[4]United States Department of Agriculture, Agriculture Research Service, retired. Current address Jaronski Mycological Consulting, Blacksburg VA 24060 USA

*allanfmarc@gmail.com

*vaniabit@gmail.com

*gmmascar@gmail.com

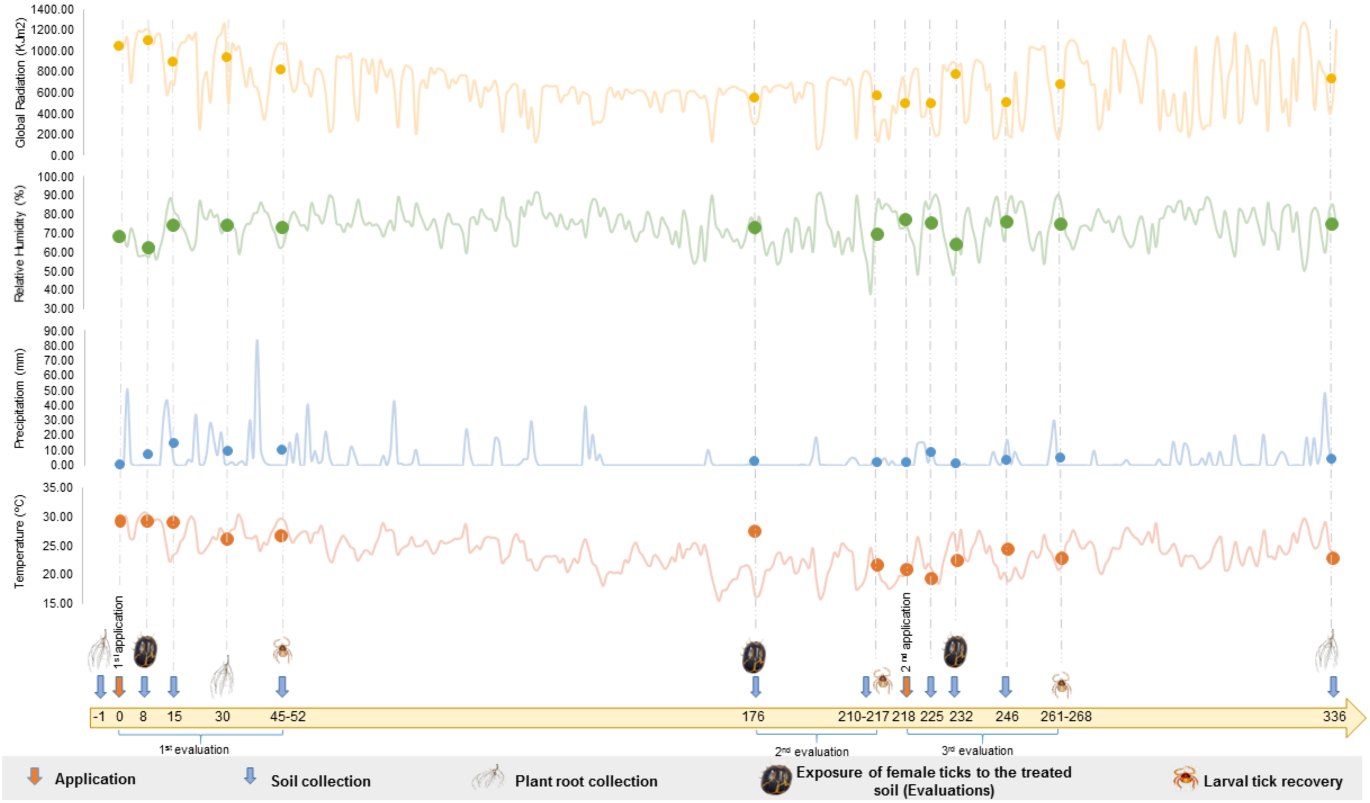

Figure S1. Timeline illustrating the days, climate periods, and environmental parameters with the days of fugal treatments, soil collections, female ticks exposures, larval tick recovery and assessment of fungal persistence through the soil and root collection over the 337 days of the test in semi-field conditions. Points indicate the average value of the climatic variable on the indicated days.

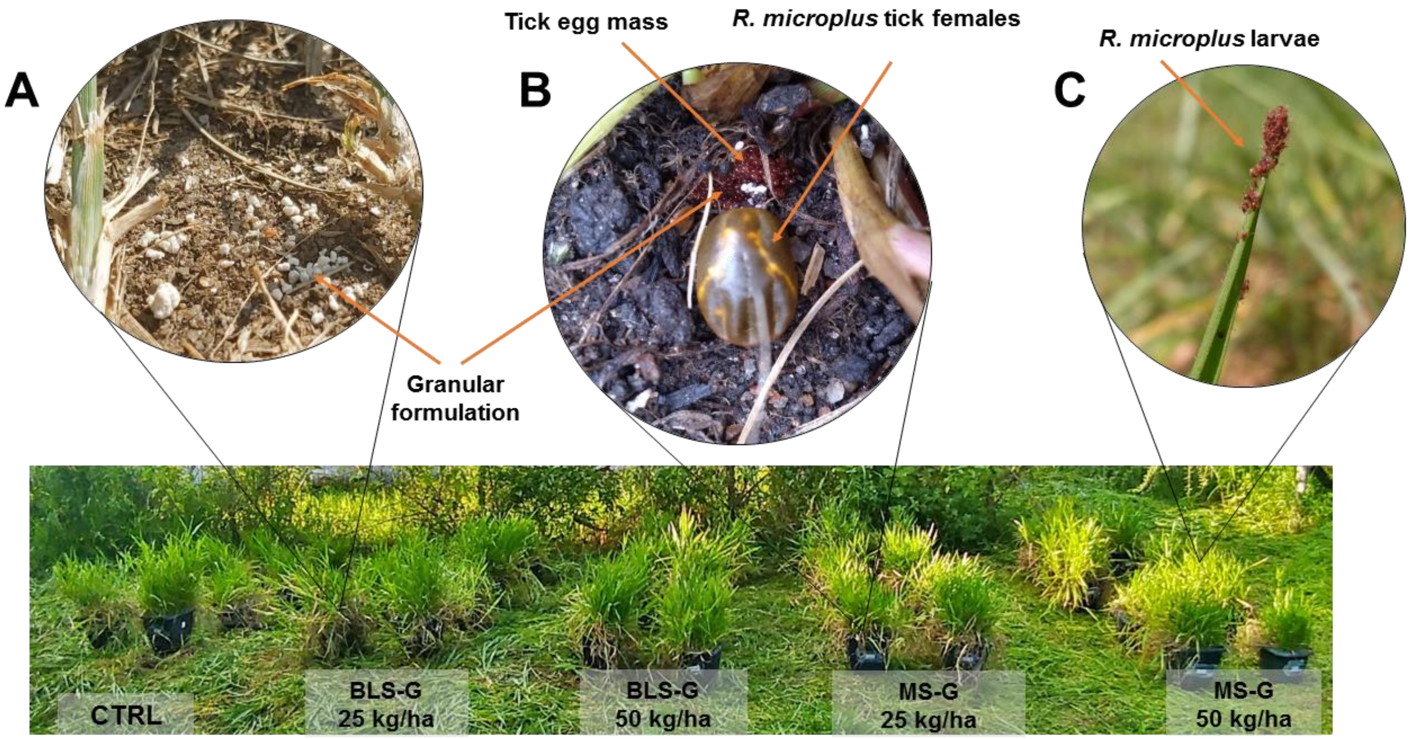

Figure S2. *Arrangement of pots containing* Urochloa decumbens *cv. Basilisk randomly distributed in the four pot groups treated with 25 or 50 kg/ha of granular formulations containing* Metarhizium robertsii *microsclerotia (MS-G) or blastospores (BLS-G) (BLS-G 25; BLS-G 50; MS-G 25; MS-G 50) and one control group (CTRL), under field conditions.* ***A)*** *Granules distributed over the soil surface of the pots;* ***B)*** Rhipicephalus microplus *engorged female on the soil of fungus-treated pot during the egg-laying period and granular formulation (white color) on the egg mass;* **C)** *Larvae on the top of the grass blade after hatching.*

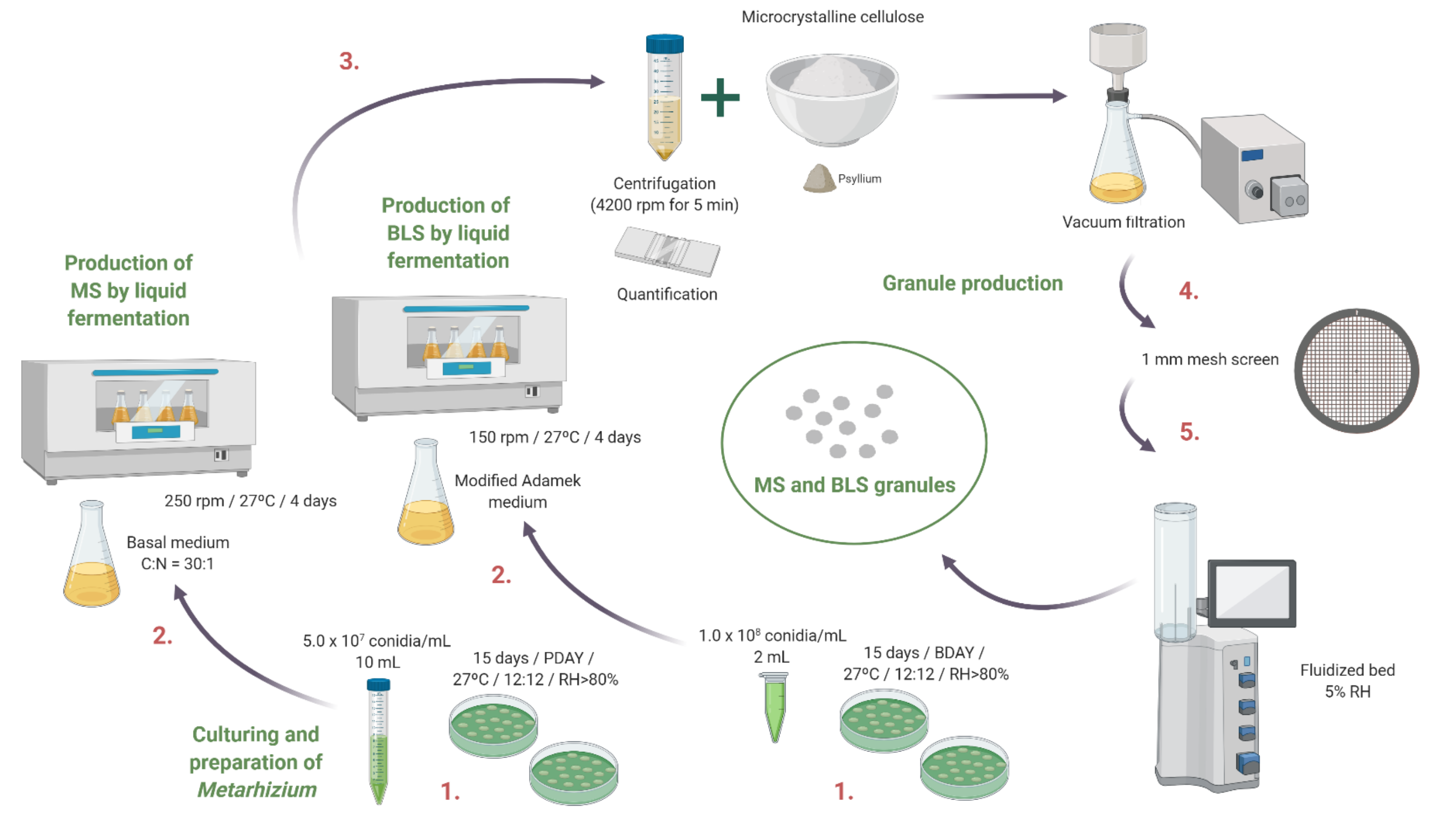

Figure S3. Illustrative step-by-step procedure for the production of *Metarhizium robertsii* microsclerotia (MS) and blastospores (BLS) granules. 1) Obtaining fungal suspensions; 2) inoculation in specific media for the production of MS and BLS; 3) Separation of formed biomass and mixing with inert ingredients; 4) Manual extrusion of the formulated material resulting in the granules; 5) Drying the ready granules in a fluidized bed dryer.

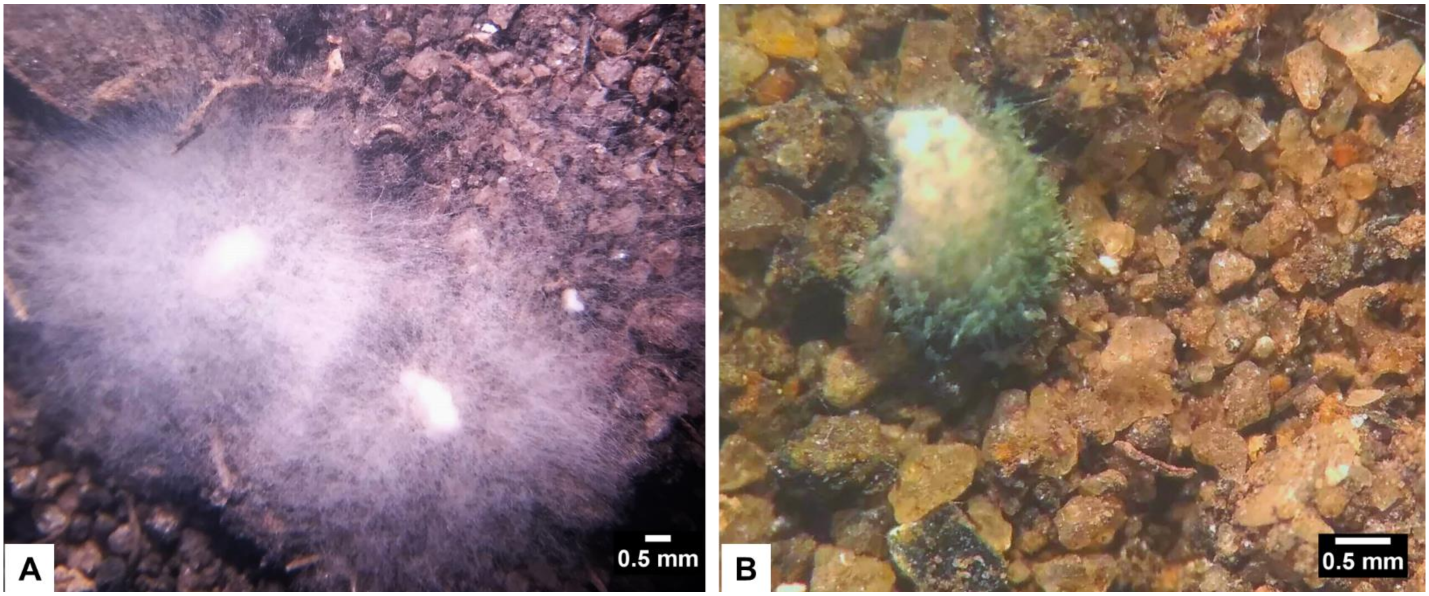

Figure S4. Hyphal germination of *Metarhizium robertsii* blastospore-based granules (A) and sporogenic germination of a microsclerotial granule visualized during the assessment of fungal viability under a stereoscope microscope. Scale bar = 0.5 mm.

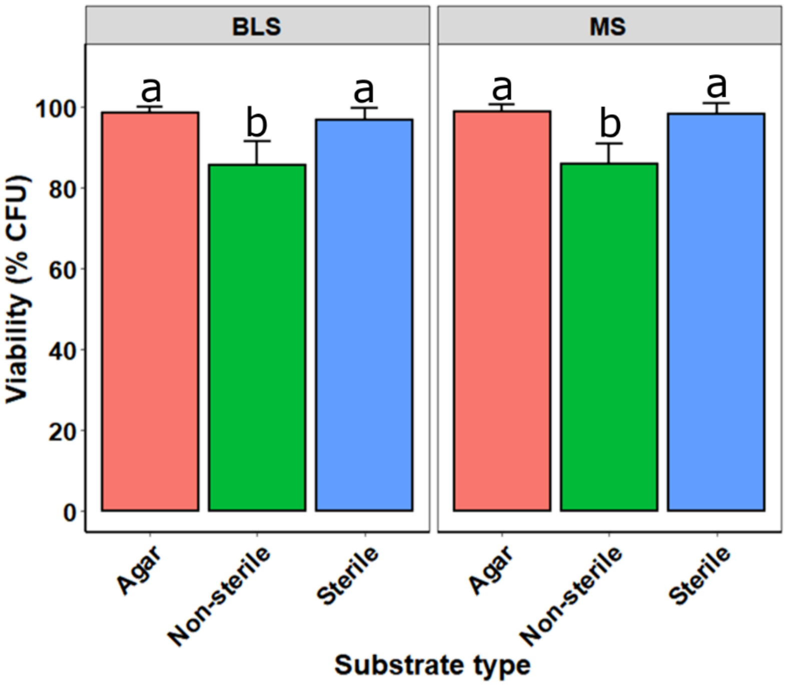

Figure S5. Conidial viability assessed by UFC count after sporogenic germination by *Metarhizium robertsii* granules upon rehydration and incubation on different substrate types (agar water, non-sterile soil and sterile soil). Vertical bars (means ± 95% CI = confidence interval) followed by distinct letters are statistically different, within each fungal propagule (MS = microsclerotia and BLS = blastospores)(Tukey HSD, $P < 0.05$).

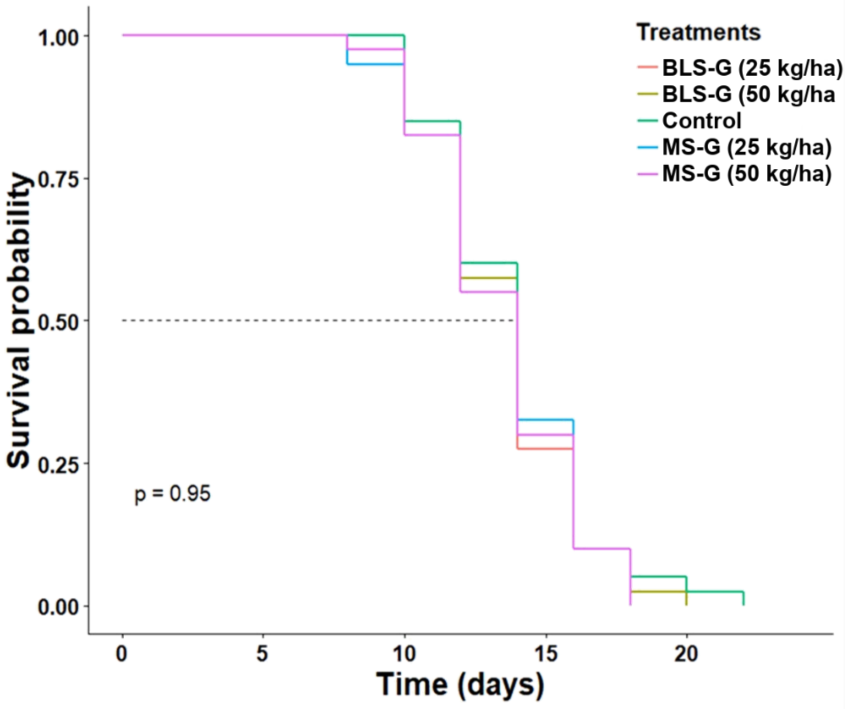

Figure S6. Probability of survival of *Rhipicephalus microplus* engorged females throughout the days after exposure to treated soil with different rates (50 and 25 kg/ha) and types (microsclerotia = MS and blastospores = BLS) of *Metarhizium robertsii* granular formulations*.* Dashed line indicated the median (50%) lethal time. *P*-value is shown and is not significant at 5% probability (Likelihood ratio test (LRT) = 0.88, df = 4, *P* = 0.95).

A) 1st Application (8 to 210 days)

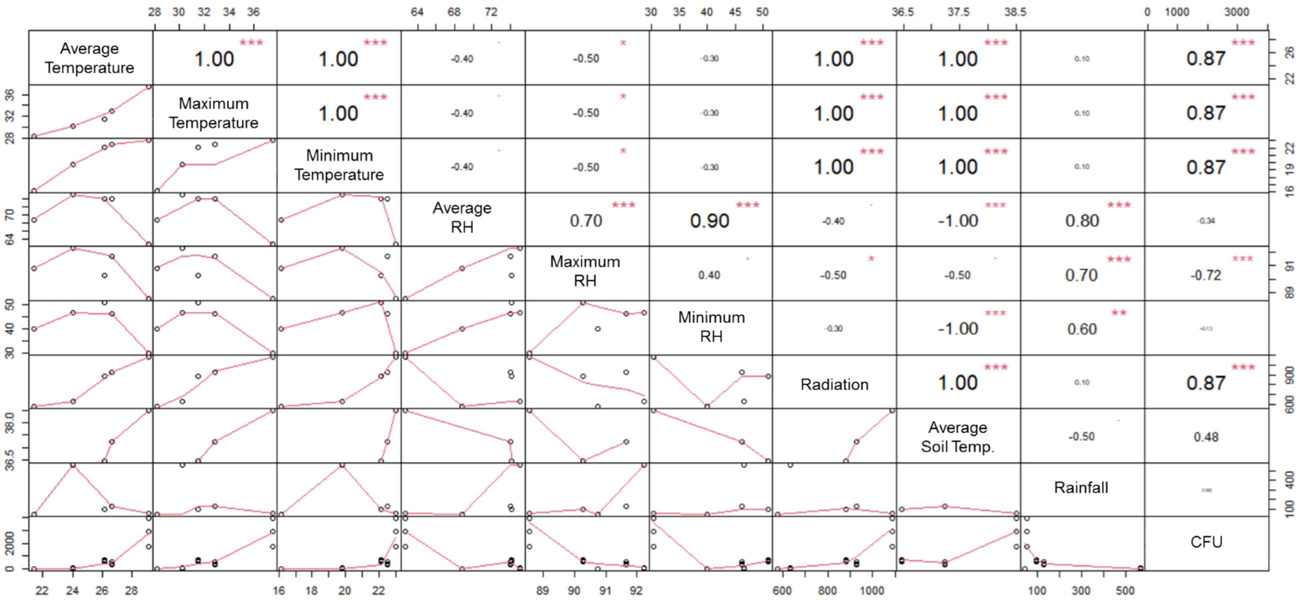

B) 2nd Application (225 to 336 days)

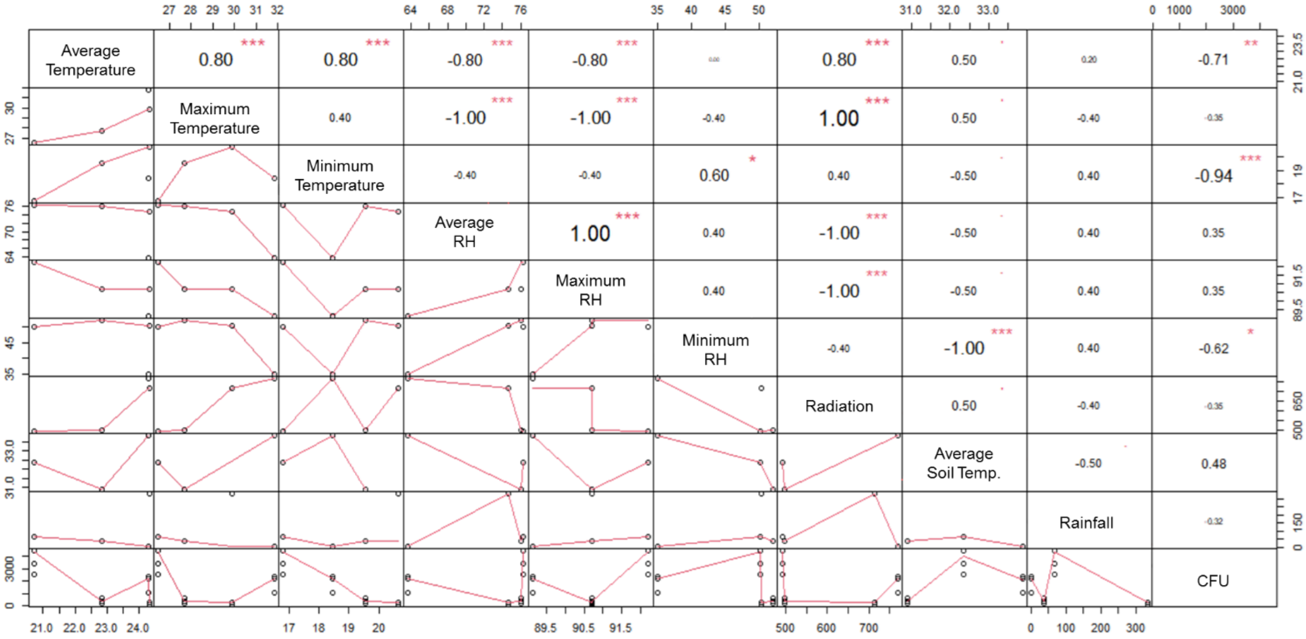

Figure S7. Spearman's correlation between the number of colony forming units (CFUs)/g soil and abiotic variables, including temperatures, relative humidity, soil temperature and solar radiation during the experimental time course. **A)** Data correlation after the first application of fungal formulations (day 8 to day 210). **B)** Data correlation after the second application of fungal formulations (day 225 to day 336).

UFRRJ
Universidade Federal Rural do Rio de Janeiro

Comissão de Ética no Uso de Animais
Instituto de Veterinária

CERTIFICADO

Certificamos que a proposta intitulada "Manutenção de colônia de Rhipicephalus microplus visando a avaliação do efeito de fungos entomopatogênicos no seu controle", protocolada sob o CEUA nº 9714220419 (ID 001419), sob a responsabilidade de **Vânia R. E. Pinheiro Bittencourt** *e equipe; Patrícia Silva Gôlo; Isabele da Costa Ângelo; Mariana Guedes Camargo; Amanda Costa da Rocha Corval; Laura Nóbrega Meirelles; Allan Felipe Marciano; Emily Mesquita da Silva; Fernanda Sousa Faria ; Ricardo de Oliveira Barbosa; Jéssica Fiorotti de Paulo; Julie Rhanna Tavares Ferreira; Thaís Almeida Corrêa* - que envolve a produção, manutenção e/ou utilização de animais pertencentes ao filo Chordata, subfilo Vertebrata (exceto o homem), para fins de pesquisa científica ou ensino - está de acordo com os preceitos da Lei 11.794 de 8 de outubro de 2008, com o Decreto 6.899 de 15 de julho de 2009, bem como com as normas editadas pelo Conselho Nacional de Controle da Experimentação Animal (CONCEA), e foi **aprovada** pela Comissão de Ética no Uso de Animais da Instituto de Veterinária da Universidade Federal Rural do Rio de Janeiro (CEUA/UFRRJ) na reunião de 30/08/2019.

We certify that the proposal "Maintenance of Rhipicephalus microplus colony aiming to evaluate the entomopatogenic effect of fungi", utilizing 3 Bovines (3 males), protocol number CEUA 9714220419 (ID 001419), under the responsibility of **Vânia R. E. Pinheiro Bittencourt** *and team; Patrícia Silva Gôlo; Isabele da Costa Ângelo; Mariana Guedes Camargo; Amanda Costa da Rocha Corval; Laura Nóbrega Meirelles; Allan Felipe Marciano; Emily Mesquita da Silva; Fernanda Sousa Faria ; Ricardo de Oliveira Barbosa; Jéssica Fiorotti de Paulo; Julie Rhanna Tavares Ferreira; Thaís Almeida Corrêa* - which involves the production, maintenance and/or use of animals belonging to the phylum Chordata, subphylum Vertebrata (except human beings), for scientific research purposes or teaching - is in accordance with Law 11.794 of October 8, 2008, Decree 6899 of July 15, 2009, as well as with the rules issued by the National Council for Control of Animal Experimentation (CONCEA), and was **approved** by the Ethic Committee on Animal Use of the Veterinary Institute of Rural Federal University of Rio de Janeiro (CEUA/UFRRJ) in the meeting of 08/30/2019.

Finalidade da Proposta: Pesquisa (Acadêmica)

Vigência da Proposta: de 09/2019 a 09/2024 Área: Parasitologia Animal

Origem:	Setor de Bovinocultura da UFRRJ						
Espécie:	Bovinos	sexo:	Machos	idade:	6 a 48 meses	N:	3
Linhagem:	Mestiços das raças Holandesa e Gir			Peso:	100 a 400 kg		

Local do experimento: Os animais serão mantidos em baias na Estação para Pesquisas Parasitológicas W. O. Neitz do Departamento de Parasitologia Animal, Instituto de Veterinária da UFRRJ.

Seropédica, 01 de outubro de 2019

Prof. Dr. Fabio Barbour Scott
Coordenador da Comissão de Ética no Uso de Animais
Instituto de Veterinária da Universidade Federal Rural do Rio de Janeiro

Carlos Alexandre Rey Matias
Vice-Coordenador da Comissão de Ética no Uso de Animais
Instituto de Veterinária da Universidade Federal Rural do Rio de Janeiro
